# Tibialis posterior tendon entrapment in posterior malleolar and pilon injuries of the ankle: a retrospective analysis

**DOI:** 10.1007/s00590-023-03714-8

**Published:** 2023-09-12

**Authors:** Junaid Aamir, Anxhela Syziu, Loukas Andritsos, Robyn Caldwell, Lyndon Mason

**Affiliations:** 1 Liverpool Orthopaedic Trauma Service, Liverpool University Hospital NHS Foundation Trust, Liverpool, UK; 2 School and Medicine, University of Liverpool, Liverpool, UK; 3 Department of Trauma and Orthopaedics, Aintree University Hospital, Liverpool, UK

**Keywords:** Tibialis posterior, Pilon fracture, Tendon entrapment

## Abstract

**Background:**

The Tibialis Posterior tendon (TPT) is the only tendon to encounter the distal tibia and is therefore at greatest risk of injury in fractures of the distal tibia. Although TPT injury has been reported rarely with injuries around the ankle, they often have been missed and present late.

**Aim:**

Our aim was to analyse the rate to TPT entrapment in fractures involving the posterior tibia, i.e. Pilon (PLM) and posterior malleolar fractures (PMF).

**Methods:**

A retrospective analysis of PMF and Pilon fractures over an 8-year period was undertaken. Patients who had undergone surgical fixation of their PMF or PLM were identified from 2014 to 2022, using our prospectively collected database. Any fracture which had undergone a preoperative CT was included. Analysis of their pre-operative CT imaging was utilised to identify TPT entrapment, where if < 50% of the tendon cross section was present in the fracture site, this was denoted as a minor entrapment and if ≥ 50% of the tendon was present in the fracture site was denoted as major.

**Results:**

A total of 363 patients were identified for further analysis, 220 who had a PMF and 143 with PLM injury. The incidence of TPT entrapment was 22% (*n* = 79) with 64 minor and 15 major entrapments. If the fracture line entered the TPT sheath, there was a 45% rate (72/172) of entrapment as compared to 3.7% (7/190) in fractures not entering the sheath (*p* < .001). There was no significant difference in TPT entrapment in PMF as compared to PML (*p* = 0.353).

**Conclusion:**

In our assessment, we found significant prevalence of 22% of TPT entrapment in fractures involving the posterior tibia. PMF and PLF had no statistically significant difference in the rate of TPT entrapment. Additionally, we found that there was a significant risk of TPT entrapment when the CT images display the fracture line entering the tendon sheath. We recommend that surgeons consider taking care assessing pre-operative imaging to seek to identify the TPT and to assess intraoperatively where entrapment does occur.

## Introduction

Posterior Malleolar fractures (PMF) and Pilon fractures (PLF) are often complex and high energy injuries which require significant surgical involvement. Pilon injuries are associated with axial loading to the ankle and surgeons who manage these often encounter significant complications pre and postoperatively with soft tissue loss/interposition, complex fracture configurations and poor functional outcomes being a few highlighted within the literature [1–3]. *Korkmaz *et al*.* highlighted that quality of the reduction was in fact the most important factor affecting outcomes in the surgical management of PLF [1]. This evidence is further supported by *Sajjadi *et al*.* who identified that anatomical reduction in PLF can assist in both soft tissue recovery and also facilitate faster rehabilitation and minimise hospital inpatient stay [4]. Posterior malleolar injuries have also been highlighted to be at risk of developing similar complications relating to obtaining adequate reduction in the fracture. Anatomical reduction in PMF have been established to improve postoperative outcomes both short and long term [5–7].

The tibialis posterior tendon (TPT) is unique in its nature as it is the only tendon in the ankle which directly articulates with the tibia [8]. It is known that often the retro-malleolar groove of the tibia, where the TPT resides within the ankle is commonly affected in PMF [9]. Given this close relationship, their involvement with bony injuries in the ankle is important to assess. TPT entrapment in ankle injuries is a rare but reported complication that is difficult to visualise on imaging and can significantly affect function outcomes postoperatively [10–12]. In the context of ankle fracture, TPT entrapment can present months to years following initial injury and has been noted in several cases to be the primary cause of mal-reduction in the ankle mortise intraoperatively [7, 13, 14]. Of note, *Eastman *et al*.* documented their review of 420 PLF injuries which identified entrapped posterior structures within the ankle in 9.5% of patients [15]. Of these patients with entrapped structures, 95% of the cases sustained a TPT entrapment and radiology reporting CT scans only noted the interposed structure 20% of the time on initial assessment [15].

Currently, there is very limited evidence of how the TPT interacts with the ankle in the context of both PMF and PLF injuries. Additionally, there is minimal evidence reviewing the postoperative outcomes of these patients that did sustain TPT entrapment. The primary aim of this study is to analyse the TPT entrapment in fractures involving the posterior tibia (PMF and PLF) the null hypothesis being that there is no association with entrapment rates and more severe PMF and PLF.

## Methods

All patients from who were admitted to Aintree University Hospital Major Trauma centre between June 2014 to August 2022 with PMF and PLF fractures were identified. Data was obtained from our institutions Bluespier electronic patient note system. We included adult patients who sustained PMF and PLF that had undergone pre-operative CT scans of their ankle and were managed operatively. Those patients who did not receive a pre-operative CT scan of their ankle or underwent non-operative management for their injury were excluded.

**Fig. 1:**
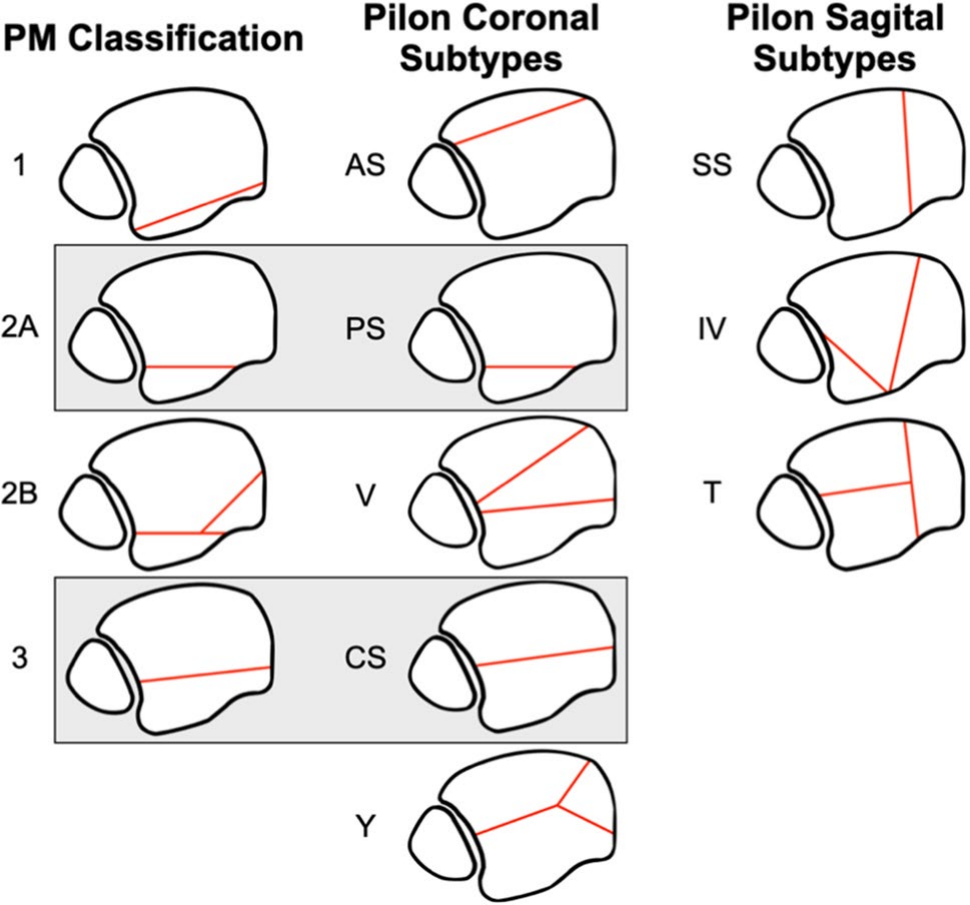
Mason–Molloy and Topliss Classification Systems utilised to determine fracture pattern [17, 18]

**Fig. 2:**
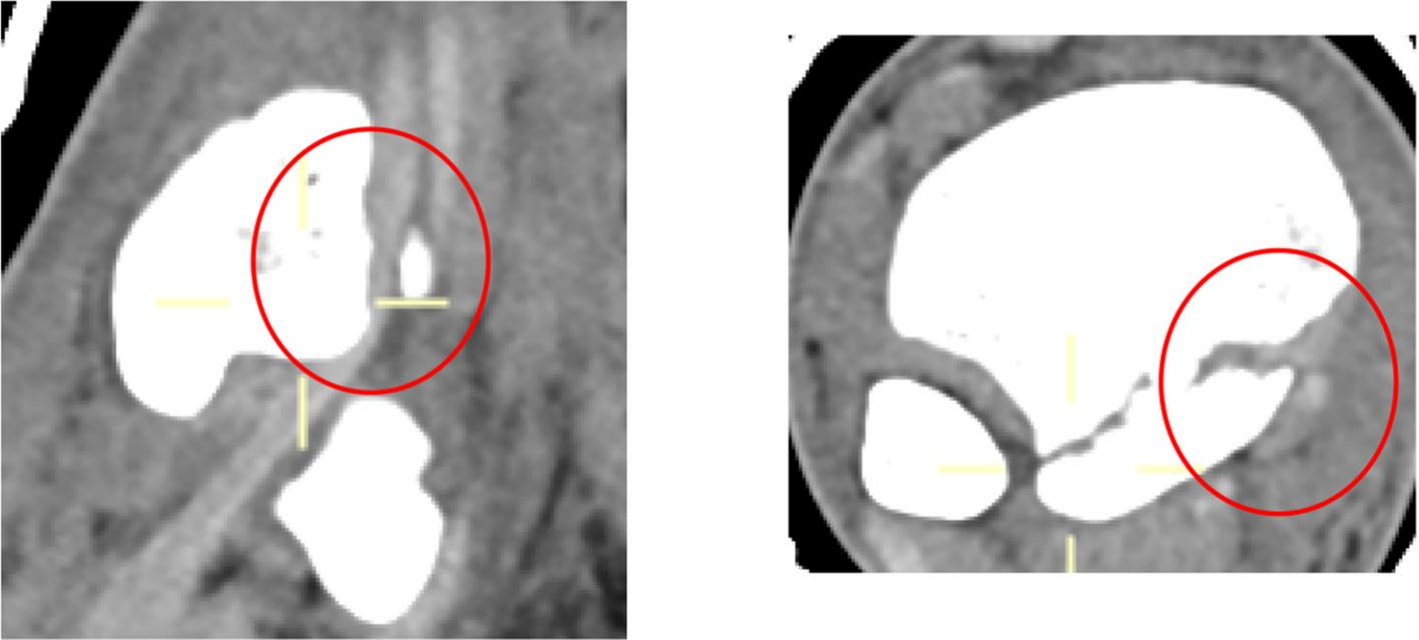
Major Tendon entrapment associated with a 2A PMF where the fracture enters the tibalis posterior sheath

**Fig. 3:**
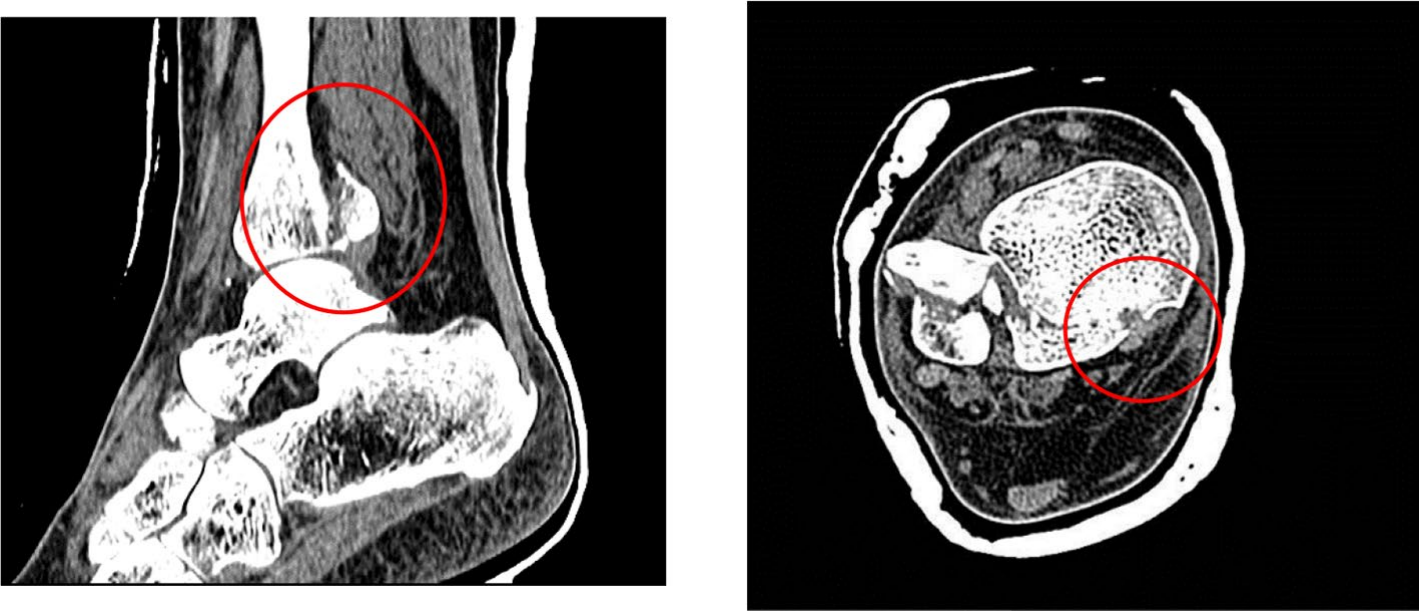
Minor Tendon entrapment associated with a 2A PMF where the fracture enters the < 50% of tibialis posterior sheath

Ankle fracture characteristics were identified from our Picture Archiving and Communication System (PACS). Analysis was performed of all preoperative, intraoperative and postoperative imaging. Data collected was specific to initial injury, Mason–Molloy classification [16] for PMF and Topliss classification for PLF [17, 18] (Fig. 1). Additionally, further information was elicited relating to whether the injury was an open fracture and if the patient sustained a dislocation of the ankle, was it adequately reduced in the first instance. Pre-operative radiology images were reviewed in their soft tissue windows to determine whether the fracture line entered the TPT sheath and whether there was visible entrapment. Entrapment of the TPT was defined as “no entrapment”, “entrapment—minor” and “entrapment—major”. The definition of minor was if < 50% of the tendon diameter was entering the fracture on axial slice CT. Major entrapment was defined as ≥ 50% of tendon diameter entering the fracture on axial slice (Figs. 2 and 3).

Intraoperative data was obtained relating to the primary surgical approach utilised (Medioposteromedial, Posteromedial, Posterolateral, Lateral, Anterior) and whether TPT entrapment has been noted. Finally, electronic patient records were assessed for whether patients complained of residual symptoms of any description at the 6 months of beyond mark within their follow-up.

Statistical analysis was performed using SPSS (IBM Corp, USA). Comparisons were made between group characteristics. Chi-squared and Fisher’s exact tests were used for categorical variables and independent samples t tests and Mann–Whitney *U* test for variable means. Uni- and multivariate analyses were performed using univariate and multivariate logistic regression analysis to identify factors involved in mortality. Any factor which achieved significance on univariate analysis underwent further multivariant regression analysis. Statistical significance was assumed when *p* < 0.05.

## Results

A total of 363 patients were identified across an 8-year period. There were 220 patients (60.6%) in the PMF group and 143 patients (39.4%) in the PLF group. The average age of patients was 49.2 years (95% CI 47.6, 50.9). Twenty-one patients (5.3%) sustained an open ankle fracture. Around 45% of patients (165/363) sustained an acute ankle dislocation, of those, 71% (117/165) were adequately reduced on initial reduction attempt in the emergency department. Regarding the PMFs, there were 69 (31.4%) type 1, 81 (36.8%) type 2A, 52 (23.6%) type 2B and 15 (6.8%) type 3 Mason and Molloy. Regarding PLF, there was 27 (18.9%) coronal split, 18 (12.6%) posterior split, 18 (12.6%) anterior split, 15 (10.5%) V-type, 28 (19.6%) Y type, 12 (8.4%) sagittal split, 9 (6.3% inverted V and 19 (13.3%) *T*-type on the Topliss classification. We note that there are some significant similarities within specific fracture pattern types between PLF and PMF; although they were categories individually within the results and analysis, we feel as though they should be grouped together when considering the clinical context of these injury types (Fig. 1).

Our total TPT entrapment rate across both PLF and PMF was 21.76% (79/363). This amounted to 64 minor entrapments and 15 major entrapments, (17.63% and 4.13% of total patients, respectively). There was also no significant difference between TPT entrapment rates amongst the PMF and PLF groups with PMF having 36 (16.36%) minor entrapments and 7 (3.18%) major entrapments and PLF having 28 (19.58%) minor entrapments and 8 (5.59%) major entrapments (*p* = 0.353). A breakdown of tibialis posterior tendon entrapment with fracture classification type is illustrated in Table 1. There was no significant difference in tendon entrapment across either the PMF fracture types or PLF fracture types.

**Table 1:** Cross tabulation of tibialis posterior entrapment with posterior malleolar fracture (PMF) classification (Mason and Molloy) and pilon fracture (PLF) classification (Topliss) (*N*—number, %—percent)

		No Entrapment		Minor entrapment		Major entrapment		Total
		*N*	%	*N*	%	*N*	%	
PMF	1	56	81.16	10	14.49	3	4.35	69
	2B	41	78.85	8	15.38	3	5.77	52
Total		174	80.18	36	16.59	7	3.23	217
PLF	AS	17	94.44	1	5.56	0		18
	V	13	86.67	2	13.33	0		15
	Y	20	71.43	6	21.43	2	7.14	28
	SS	7	58.33	3	25.00	2	16.67	12
	iV	6	66.67	2	22.22	1	11.11	9
	T	12	63.16	6	31.58	1	5.26	19
Total		110	75.34	28	19.18	8	5.48	146
	PMF 2A and PLF PS	82	82.82	17	17.17	0		99
	PMF 3 and PLF CS	30	71.43	9	21.43	3	7.14	42
Total		111	79.29	26	18.57	3	2.14	140

Our analysis of the CT scan displayed that if the fracture entered the TPT sheath, there was a prevalence of 41.86% (72/172) TPT entrapment, compared to 3.7% (7/190) of fractures that did not enter the sheath. (*p *< 0.001) (Table 2). Both univariate and multivariate regression analysis was used to identify any other factor that may predispose to TPT entrapment (Table 3). On final multivariate regression analysis, only the fracture line entering the tibialis posterior remained significant in association with TPT entrapment (OR 18.44, 95% CI 8.17, 41.63).

**Table 2:** Cross tabulation of fracture line entering tibialis posterior tendon (TPT) sheath and tibialis posterior tendon entrapment. (*N*—number, %—percent)

	No entrapment		Minor entrapment		Major entrapment		Total
	*N*	%	*N*	%	*N*	%	
Fracture line entering TPT Sheath	100	58.14	59	34.30	13	7.56	172
Fracture line NOT entering TPT Sheath	183	96.32	5	2.63	2	1.05	190
*p* Value	< 0.001

**Table 3:** Univariate and multivariate analysis of factors affecting TPT entrapment including posterior malleolar fracture (PMF) classification (Mason and Molloy) and Pilon fracture (PLF) classification (Topliss)

Factor	Univariate				Multivariate			
		OR	95% C.I			OR	95% C.I	
			Lower	Upper			Lower	Upper
PMF	1	0.836	0.871	0.234	3.237			
	2B	0.262	1.982	0.599	6.555			
Pilon	AS	0.187	5.081	0.454	56.813			
	V	0.407	2.254	0.33	15.369			
	Y	0.978	1.021	0.243	4.297			
	SS	0.394	0.465	0.08	2.701			
	iV	0.888	1.146	0.173	7.608			
	T	0.582	0.684	0.177	2.642			
Combined	2A and CS	0.764	1.223	0.328	4.558			
	3 and PS	0.628	1.374	0.38	4.967			
Open	0.815	1.132	0.401	3.191				
Dislocation	0.071	1.586	0.961	2.618	0.287	1.359	0.773	2.388
Reduced	0.133	1.487	0.886	2.495				
Tib Post sheath	** < .001**	**18.823**	**8.345**	**42.454**	** < .001**	**18.442**	**8.169**	**41.632**

Bold type marks significance

Further analysis of fracture lines displayed that Topliss T-type (8%, *p* = 0.019), Topliss Coronal split (10.5%, *p* = 0.038) and Mason-Molloy type 1 (9.8%, *p* < 0.001) fracture lines entered the TPT tendon sheath at the highest rates which achieved statistical significance. Postoperatively, approximately 24% of patients complained of residual symptoms in the ankle up to and beyond the 6-month mark (94/363). Repeat CT imaging was performed in approximately 10% of cases following initial fixation (43/363). TPT entrapment was identified in 18% of cases that underwent follow-up CT scan, 6 minor (13.9%) and 2 major (4.65%).

## Discussion

The primary aim of this study was to analyse the TPT entrapment in fractures involving the posterior tibia (PMF and PLF) with the null hypothesis being that there is no association with entrapment rates and more severe PMF and PLF. We found that the prevalence of entrapment was almost 22% in fractures involving the posterior tibia; however, there was no particular fracture classification type which entrapment was significantly associated with. One factor identified as important, however, was the fracture line entering the TPT sheath, which was significantly involved with TPT entrapment regardless of fracture type.

Over an 8-year period, a total of 363 patients were identified with either a PLF or PMF. We have identified a 45% risk of TPT entrapment when the fracture line enters the tendon sheath on axial CT images. Currently, we have found no studies which can corroborate this finding, and this suggest that pre-operative CT scans may provide the clue into fully assessing the risk of TPT entrapment in patients with either PMF or PLF. Other factors such as open fracture or dislocation on admission did not contribute to TPT entrapment rates in this study. Postoperatively we found that 18% of patients who underwent follow-up CT imaging still had some element of entrapment after surgical fixation; however, the TPT entrapment had not been looked for historically and would not have been planned to be cleared from the fracture site at time of surgery.

To this date, the TPT entrapment in trauma cases is a topic that has very little published research. Our assessment of both PLF and PMF is supported by other current evidence [10, 12, 15, 19–21]. A study by Eastman et al. analysing 420 pilon fractures found 36 TPT entrapments on CT scan also supported on their conclusions the importance of pre-operative CT scan in diagnosing the entrapment and assisting in the surgical planning of those cases [15]. Ballard et al. analysed 398 patients and found a prevalence of 90% TPT entrapment in 30 patients with entrapped tendons in either pilon or ankle fractures recommending preoperative CT scan for these injuries and adding the risk of concurrent FHL and TPT tendons in few of these cases [21]. *Sousa *et al*.* discovered in a retrospective analysis that PLF Ruedi–Allgower type 2/3 were 8 times more prone to tendon injury, particularly TPT entrapment. (*p* = 0.005; OR 7.5; 95% CI 1.72–32.80) [19, 22]. This evidence is also supported by *Cardoso *et al*.* who established that TPT injury was most common in PLF, with entrapment being the most common lesion [20]. Their functional assessment at 2-years follow-up, however, did not display any differences between those who has sustained a TPT injury and those who had not. (*p* = 0.281) [20] There is currently a lack of evidence comparing the entrapment rates between PMF and PLF groups. Our study has shown that the TPT entrapment rates are comparable in PMF with PLF.

Upon review of postoperative outcomes, we identified that almost a quarter of patients had residual symptoms up to or beyond the 6 months follow-up. Although, we did not formally undertake patient reported outcome measures, we did not find a significant association between type of entrapment and presence of postoperative symptoms (*p* = 0.227). Considering the potential injury to the TPT if entrapped, surgical planning to clear the TPT from the fracture site should be considered when undertaking surgery. Both Philpott et al. and Gandham et al. describe the medial posterior medial approach to the posterior tibia where the TPT is identified and can thus be used in such cases [18, 23].

## Limitations

The retrospective nature of the study resulted in gaps within analysis, particularly the follow-up assessment of patients out of the area, who were operated locally, was not able to be consistent. Also, the majority of the clinic letters did not specifically document on TPT symptoms and the lack of CT scans postoperatively made difficult to identify and analyse TPT entrapment cases that may have resolved conservatively or with physiotherapy.

## Future directions

Our study sets a basis for future research on the subject. Other factors may have been associated with TPT entrapment which have not been assessed. Larger patient cohorts may be able to delineate the correlation between the fracture pattern and the risk for TPT entrapment to aid surgeons’ decision regarding what surgical approach they use. Prospective follow-up of patients with TPT entrapment may help to identify TPT specific function which our retrospective study cannot assess. Similarly, post-surgical CT may also be used to identify if TPT clearance has been successful. Lastly, we have introduced a novel grading system on TPT entrapment that can help in the management of such cases postoperatively. Further assessment of our grading system is required to evaluate its clinical correlation in terms of symptoms and management which can by standardised for ‘minor’ and ‘major’ entrapment cases.

## Conclusion

Overall, our retrospective review displayed that there was a significant risk of TPT entrapment when CT images display the fracture line entering the tendon sheath. Additionally, that there was no significant difference in entrapment rates between PML and PMF. We recommend that surgeons consider pre-operative CT scan in all pilon and posterior malleolar fractures and seek to identify the TPT to determine whether there is a risk of entrapment. To our knowledge our study is the first one to quantify the TPT entrapment grade. Further analysis should be performed to investigate factors contributing to TPT entrapment postoperatively and any relation to a ‘minor’ or ‘major’ entrapment in the initial CT scan and also, functional outcomes of patients following this.

## Abbreviations

TPT: Tibialis Posterior Tendon
PMF: Posterior Malleolar Fractures
PLM: Pilon Fractures
CT scan: Computed Tomography scan
FHL: Flexor Hallucis Longus
